# Valuing the years of life lost due to COVID-19: the differences and pitfalls

**DOI:** 10.1007/s00038-020-01430-2

**Published:** 2020-07-20

**Authors:** Brecht Devleesschauwer, Scott A. McDonald, Niko Speybroeck, Grant M. A. Wyper

**Affiliations:** 1 Department of Epidemiology and Public Health, Sciensano, Brussels, Belgium; 2 Department of Veterinary Public Health and Food Safety, Ghent University, Merelbeke, Belgium; 3 Centre for Infectious Disease Control, National Institute for Public Health and the Environment (RIVM), Bilthoven, The Netherlands; 4 School of Public Health and Research Institute of Health and Society, Catholic University of Louvain, Brussels, Belgium; 5 Place and Wellbeing Directorate, Public Health Scotland, Glasgow, Scotland, UK

The only thing that is certain about death is that upon it, no life remains, and that the risk of death during a person’s lifetime is 1. These facts cannot be disputed; however, assessments over how much life has been prematurely lost upon death have led to polarised views. The impact of COVID-19 is drawing increased attention on how we approach putting a value on the life prematurely lost by death (Appleby 2020; Hanlon et al. 2020; Kirigia and Muthuri 2020).

Years of life lost to premature mortality (YLL) is a frequently used population health metric, originating back to the 1940s (Haenszel 1950). The idea is appealingly simple—instead of merely counting the number of deaths, each death is weighted as a function of the age at death, reflecting the common appreciation that deaths at young ages are more severe than deaths at advanced ages. However, there is no single unique way to operationalise the concept, reflecting the reality that YLL can never be observed. Indeed, the estimation of YLL requires assumptions on the counterfactual, parallel world that did not happen—how long would the person have lived had they not have died?

The debate around this normative assumption is largely centred on the choice of mortality risk that residual values for age-conditional life expectancy in YLL calculations are based on. Should they be based upon mortality risks that are country-specific, or risks that are external to the population studied, and are chosen to be aspirationally low? It may seem rational to use national life tables, reflecting the country-specific mortality risks, until we estimate residual life expectancy for sub-national units. This highlights that particular groups, such as those with a socioeconomic disadvantage, have very different mortality risks. Take Singapore, which has the highest life expectancy in the world (Institute for Health Metrics and Evaluation. GBD results tool. Global Health Data Exchange 2020). The mortality risk in Singapore is not representative for that in Scotland—for instance, the former country has a residual life expectancy for females aged 75 that is 3.67 years higher than the latter. However, looking at differences between the most and least deprived areas for this demographic in Scotland also yields a large disparity, of 2.91 years (National Records of  Scotland 2016). This raises the issue of why people are comfortable with the idea that life could be valued differently between countries, but are then uncomfortable with the idea of assigning different values of residual life expectancy on the basis of an individual’s sub-national location. Using a national life table furthermore creates a paradox by which increased mortality risks, of for instance the COVID-19 pandemic, could cause life expectancy to go down, which could result in a contradictory reduction in estimates of YLL (McCartney et al. 2020).

A second major point of discussion is whether YLL should be corrected for comorbidities of the deceased (Hanlon et al. 2020; Cassini et al. 2019). This is particularly the case for COVID-19, which frequently causes death in the old and frail, and those with underlying chronic conditions. Some thus argue that valuing the death of a 90-year-old nursing home resident with advanced cardiac decompensation using the national life expectancy for 90-year olds would “overestimate” YLL due to COVID-19.

What these discussions make clear is the importance of transparency in documenting the exact method used to calculate YLL. Since YLL cannot be observed, they can only be estimated, and obviously, the choice of counterfactual will have a major influence on the resulting estimates. Conversely, YLL can never be “overestimated” or “underestimated”, since there is no “true” value of YLL.

The paradoxes and pitfalls described here can be circumvented by using a “standard” life table, based on aspirational mortality risks. Although these mortality risks may be lower than are currently observed in countries, they have many comparative and ethical advantages. This approach ensures that we do not accept a level of mortality risk merely because we are used to it, as to do this means we lose focus of the factors and environment that are responsible for it. Importantly, assessments on the value of human life are equal between, and within, countries. This is important as it means we are upfront about the extent of national and global inequalities, and the World Health Organisation’s goal of health for all and what that means (World Health Organisation 2020). Finally, through assuming a counterfactual based on a world free of disease, standard life tables allow measuring the impact of different diseases at the same level, which is essential for comparative studies such as the Global Burden of Disease study (GBD 2017 DALYs and HALE Collaborators 2018).
